# A Double-Blind Randomised Controlled Trial of Prebiotic Supplementation in Children with Autism: Effects on Parental Quality of Life, Child Behaviour, Gastrointestinal Symptoms, and the Microbiome

**DOI:** 10.1007/s10803-024-06239-z

**Published:** 2024-01-31

**Authors:** Jacqueline K. Palmer, Jolieke C. van der Pols, Karen A. Sullivan, Heidi M. Staudacher, Rebecca Byrne

**Affiliations:** 1 School of Exercise and Nutrition Science, Faculty of Health, Queensland University of Technology, Brisbane, QLD Australia; 2 School of Psychology and Counselling, Faculty of Health, Queensland University of Technology, Brisbane, QLD Australia; 3 Food & Mood Centre, School of Medicine, Barwon Health, IMPACT Institute, Deakin University, Geelong, VIC Australia

**Keywords:** Prebiotic, Microbiome, Autism, Quality of life, Social behaviour, Mealtime behaviour, Gastrointestinal symptoms

## Abstract

**Purpose:**

Modifying gut bacteria in children with autism may influence behaviour, with potential to improve family functioning. We conducted a randomised controlled trial to assess the effect of prebiotics on behaviour, gastrointestinal symptoms and downstream effects on parental quality of life.

**Method:**

Children with autism (4-10yrs) were randomised to 2.4 g/d of prebiotic (GOS) or placebo for six weeks. Pre and post stools samples were collected, and validated questionnaires used to measure change in social and mealtime behaviours, GI symptoms and pQOL. Linear mixed models evaluated group differences for behavioural variables, and Mann Whitney U tests were used to compare change between-groups for GI symptoms, differential abundance of genera and alpha diversity of the microbiome.

**Results:**

Thirty-three parent-child dyads completed the trial. No group difference was seen for behavioural variables but both groups improved significantly from baseline. There was a medium effect size between groups for GI symptoms (d = 0.47) and pQOL (d = 0.44) driven by greater improvements in the prebiotic group. Bifidobacterium increased threefold following prebiotics (1.4–5.9%, *p* < 0.001) with no change in controls. Supplements were well tolerated, compliance with dose 94%.

**Conclusion:**

Prebiotics modify levels of *Bifidobacterium* and prove well tolerated but in this instance, resulted in only marginal effects on GI symptoms and pQOL. A larger sample of children with more severe symptoms could help to determine the potential of prebiotics in autism.

**Trial Registration:**

https://www.anzctr.org.au/Trial/Registration/TrialReview.aspx?ACTRN=12619000615189.

## Introduction

Autism Spectrum Disorder (ASD, autism) is a neurodevelopmental disorder, now affecting up to 1 in 44 children ([CDC], 2020). It is characterised by impairments to social communications and interactions, a narrow scope of interests, repetitive behaviours and frequently, unusual sensory response (American Psychiatric Association, 2013). Parents of children with autism consistently report lower quality of life compared to parents of children without autism (Dey et al., 2019) or children with other conditions (Dabrowska & Pisula, 2010; Vasilopoulou & Nisbet, 2016) and a major contributing factor is the impact of frequent and unpredictable behavioural problems (McStay et al., 2014). Furthermore, disproportionate anxiety in the face of unfamiliar settings and routines is common, adding complexity to daily routines such as mealtimes and social occasions.

Core traits such as restrictive and repetitive behaviours (Bitsika & Sharpley, 2017) and sensory dysregulation (Chistol et al., 2018) are thought to play a role in the child’s anxious behaviours, and increase the difficulties experienced on these occasions. Children with autism are five times more likely to face difficulties around eating and mealtimes compared to children without autism (Sharp et al., 2013) and not only are their own food choices restricted, but often those of other family members (Ausderau & Juarez, 2013). Mealtimes can be particularly challenging due to their high frequency and while many stressful situations can be avoided, mealtimes represent a necessary part of child growth and development and play an important role in family connectedness (Hamilton & Hamilton Wilson, 2019). Furthermore, mealtimes are often associated with social interactions. Due to difficulty integrating their child’s needs and behaviours with social norms and expectations parents report withdrawing from social settings (Kuhlthau et al., 2014) and consequently, experience increased social isolation which is a strong predictor of lower parental quality of life (PQOL).

In addition to the behavioural aspects of mealtimes, almost half of children with autism have at least one chronic gastrointestinal (GI) symptom with constipation, diarrhoea and abdominal pain being the most frequent (Holingue et al., 2018), potentially exacerbating aberrant behaviour. GI symptom severity has previously been strongly correlated with ‘autism severity’ (Adams et al., 2011; Parracho et al., 2010). While a recent systematic review found that correlation between GI symptoms, autism severity and challenging behaviours was at times conflicting, there is evidence of some causal relationships which support the existence of gut- immune-brain pathways (Leader et al., 2022). The gut microbiome has been a topic of interest in many neurological disorders including autism, due to convincing evidence of bidirectional gut-brain communication in animal models (Bravo et al., 2011; Sudo et al., 2004). Modifying the microbiome of mice led to better coping mechanisms in the face of stress (Bercik et al., 2011), but translating outcomes of preclinical trials to humans has proven challenging (Kelly et al., 2016).

To date there is no distinct microbiome signature for autism, however low microbial diversity and a low abundance of Bifidobacterium are consistent findings (Adams et al., 2011; Coretti et al., 2018; De Angelis et al., 2013; Finegold et al., 2010; Parracho et al., 2005; Tomova et al., 2015; Wang et al., 2011). Some evidence suggests this is related to the typical low dietary diversity seen in autism, rather than with autism per se (Yap et al., 2021). However other research has shown that modifying the gut microbiome can lead to significant improvements in behaviour and in gastrointestinal symptoms in children with autism (Kang et al., 2017) with sustained effect (Kang et al., 2019). Although it is not clear what role the microbiome may play in autistic behaviours, there is growing evidence targeting the microbiome as a novel avenue for treating some of the symptoms of neurodevelopmental and psychiatric disorders.

The International Society for Probiotics and Prebiotics (ISAPP) defines prebiotics as “a substrate that is selectively utilized by host microorganisms conferring a health benefit” (Gibson et al., 2017). Prebiotics stimulate the growth of commensal bacteria which ferment substrate to produce beneficial metabolites. Major end products of prebiotic utilisation by the microbiome are short chain fatty acids that have diverse physiological roles. The most well-documented prebiotics are galactooligosaccharides (GOS) and fructooligosaccharides (FOS), which are highly bifidogenic (Gibson et al., 2017). GOS supplementation in mice led to reduced anxious behaviours (Burokas et al., 2017; Savignac et al., 2015, 2016), reduced anti-social behaviours (Szklany et al., 2020) and improved attention (Gronier et al., 2018), behaviours of interest in autism. In children, GOS has been shown to improve gastrointestinal symptoms, specifically constipation, without side-effects (Beleli et al., 2015) and in adults, GOS (but not FOS) was shown to reduce waking cortisol, an accepted marker of baseline stress (Schmidt et al., 2015). While prebiotics are naturally abundant in many foods (lentils and legumes, wholegrains, fruits and vegetables), avoidant and restrictive eating behaviours mean that these foods are often absent in the diet of children with autism (Sharp et al., 2013) and limiting substrate directly impacts the diversity of the microbiome (Yap et al., 2021).

There are a limited number of microbiome targeted interventions in children with autism and most use a probiotic (Arnold et al., 2019; Grossi et al., 2016; Liu et al., 2019; Parracho et al., 2010; Santocchi et al., 2016; Tomova et al., 2015). Prebiotics are of interest because unlike the transient effect of probiotics, they offer an opportunity for longer lasting impacts on endogenous microbiota. To date, only three microbiome targeted interventions using a prebiotic alone (GOS) (Grimaldi et al., 2018) or a combination of a prebiotic (FOS or bovine colostrum) and a probiotic (Sanctuary et al., 2019; Wang et al., 2020) have been published. Each of these interventions were small (*N* = 26, *N* = 8 and *N* = 30 respectively) but showed small improvements in anti-social behaviour and GI symptoms. These improvements in child symptoms have the potential to impact parental QOL. Therefore, the primary aim of this study was to evaluate whether supplementing the diet of children with autism aged 4–10 years with a prebiotic (GOS), could improve parental QOL (pQOL) compared with placebo. The effect of GOS on child social behaviours, mealtime behaviour and GI symptoms, and the impact on microbiome composition was also evaluated.

## Methods

### Study Design

This was a pilot six-week, double-blind, randomised, placebo-controlled trial investigating the effect of 2.4 g/d GOS (GOSYAN® GOS) supplementation in children with autism. The study was conducted from May 2019 to May 2020 in Brisbane, Australia, and was approved by the Queensland University of Technology University Human Research Ethics Committee (no. 180000074). It was prospectively registered with the Australia New Zealand Clinical Trials registry ACTRN = 12619000615189. Written consent to participate was obtained from all study participants prior to enrolment.

### Study Participants and Study Visits

Parent-child dyads with children with an existing diagnosis of autism and aged between 4 and 10 years, were recruited via paediatricians, community groups and a state-wide autism association. Children were excluded if they had taken antibiotics or antifungal medicines within the previous three months, prebiotic or probiotic supplements within the previous month, or if they had changed type or dose of medications prescribed for behaviour, anxiety, or GI symptoms, within the previous two months. No exclusions applied to parents.

**Fig. 1:**
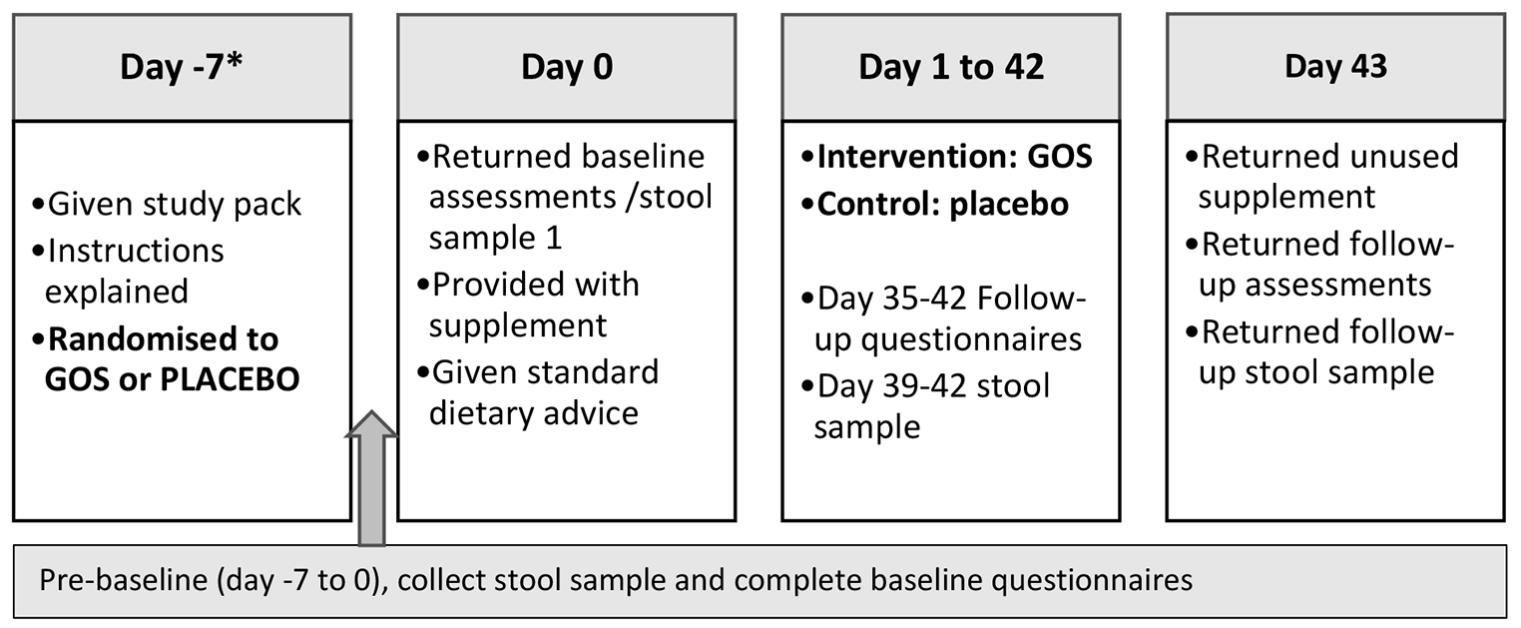
Intervention timeline

At an initial appointment parents received a study pack containing an instruction booklet, supplement diary, and two booklets (baseline and follow-up) compiled of questionnaires and records pertaining to mealtime and social behaviours, pQOL, GI symptoms and dietary intake (Fig. 1). Two Fecol® stool collection kits were included to collect stool samples from children prior to baseline and follow-up. At the baseline visit (day 0), parents returned a completed booklet of questionnaires and records and an initial stool sample and were provided their child’s allocated supplement (GOS or placebo). They also received standardised dietetic advice by a dietitian experienced in restrictive eating behaviours in children with autism (JKP) to mimic a community-based dietetic appointment. This was offered in recognition of each participants contribution to the research,. Six weeks later, parents returned a follow-up booklet of questionnaires and records that included a supplement diary, as well as a follow-up stool sample and any unused product.

### Randomisation

Participants were randomly assigned to 2.4 g/d prebiotic (GOSYAN® GOS, QHTbio) or 2.4 g/d placebo (maltodextrin D28, Manildra®) using a random block schedule, prepared by a statistician independent of the research. Labelling of the product was completed by a pharmacist independent of the research. All researchers and participants involved in the study were blinded to the allocation and only unblinded once analysis was complete.

### Intervention

The supplement/placebo was provided in capsule form with advice to commence the intervention at 1.2 g/day (2 capsules) for one week, increasing to 2.4 g/d (4 capsules) for the final five weeks. Parents were advised to mix the contents of the capsules in 50mls of a preferred liquid and offer it to their child under supervision. They were instructed to record dose consumed in the supplement diary provided. The packaging of GOS and placebo capsules were identical. Dietary advice was based on the Sequential Oral Sensory (SOS) approach to Feeding (Toomey & Ross, 2011) and simple mealtime strategies such as family eating together, and avoidance of pressuring the child to eat (Satter, 2007). The timeline for the intervention is depicted in Fig. 1.

### Assessments

#### Parental Quality of life (pQOL)

The Quality-of-Life Autism (QoLA) questionnaire (Eapen et al., 2014) was used to assess pQOL, the primary outcome for this study. This questionnaire is designed to assess the impact of caring for a child with autism and has been validated in this population (Eapen et al., 2014). The 48-item questionnaire uses a recall period of four weeks with excellent internal consistency (α > 0.9) and strong concurrent validity with all subscales of the WHOQOL-Bref (The WHOQOL Group, 1998). Responses are made using a 5-point Likert scale. The QoLA has two parts, with possible scores of 28 to 140 for part A which assesses pQOL based on factors such as self fulfillment, community support and financial security; and possible scores of 20 to 100 for Part B, which assesses the impact of the child’s autistic behaviours and traits on pQOL.

#### Child Social Behaviour

The Social Responsiveness Scale, 2nd edition (SRS-2) (Constantino & Gruber, 2012) was used to assess changes in the child’s social behaviour. This scale is sensitive to subtle change and can be used to monitor treatment progress. The 65-item questionnaire uses a recall period of six weeks, assessing ability to interact socially based on constructs of social communication, awareness, cognition, motivation, restricted interests and repetitive behaviours. Responses are made using a 5-point Likert scale and raw scores converted to T scores, categorised as mild (< 65), moderate (66–75), or severe (> 75) deficits in the ability to interact socially.

#### Child Mealtime Behaviour

The Brief Autism Mealtime Behaviour Index (BAMBI) (DeMand et al., 2015) was used to assess changes in mealtime behaviours and the difficulties that these behaviours represent for parents. The 15-item questionnaire uses a recall period of six weeks and includes three constructs based on food refusal, rigid needs and disruptive behaviours. Responses are made using a 5-point Likert scale with higher scores equating to greater dysfunction in mealtime behaviour. A cut-off score of 34 (from a possible 15–75) is used, above which behaviour is considered disruptive (DeMand et al., 2015).

#### Gastrointestinal Symptoms and Stool Output

The 6-item Gastrointestinal Severity Index (6-GSI) was used to assess change in gastrointestinal (GI) symptom severity. It measures the most common GI symptoms reported in children with autism including diarrhoea, constipation, flatulence, bloating and abdominal pain (Adams et al., 2011). The 6-item (12 point) questionnaire uses a recall period of six weeks. A score of three and above is considered in the severe range of symptoms (Adams et al., 2011). The Bristol Stool Form Scale (Heaton et al., 1992) was used to record stool consistency and stool frequency over a period of seven days prior to each visit.

#### Microbiome

Parents were provided with instructions on collection of a stool sample in the safety of the home environment using the Fecol® collection kit. A small sample of stool was collected, frozen, and stored until the research visit. Parents transported the sample on dry-ice sachets provided (Technic-Ice) and samples then transferred to cold storage (-18 °C) for batch analysis. Parents were asked to collect the sample as close to the research visit as possible, aiming for within three days of the corresponding visit.

DNA extraction was performed using the DNEasy Powersoil Kit (Qiagen, Germany) according to manufacturer’s instructions. Cell lysis was undertaken via chemical and mechanical homogenisation using a beadbeater at 3770 rpm for one minute, followed by centrifuge at 10,000 rpm to obtain a supernatant. The supernatant was cleaned, filtered and eluted with PCR grade water to collect the DNA. The concentration of DNA was measured using an Implen N60 nano photometer and samples stored at -20 °C. Library preparation (PCR amplification of V3 and V4 regions, addition of sequencing adaptors and barcode) and sequencing was completed by the Central Analytical Research Facility (QUT, Brisbane Australia), according to the Illumina 16 S guide (https://support.illumina.com/documents/documentation/chemistry_documentation/16s/16s-metagenomic-library-prep-guide-15044223-b.pdf). DNA was assayed on a Qubit 4.0 to measure concentration, then run on the Agilent fragment analyser 5200 (capillary electrophoresis) to verify amplicon size. Amplicons were sequenced on an Illumina Miseq Instrument using Miseq v3 600 cycle over 66 h. Demultiplexed sequencing results were uploaded to ‘Basespace’ and microbiota pattern analysis completed using the ‘Greengenes’ database (http://greengenes.lbl.gov/). The resulting BIOM table was uploaded to Calypso v 8.84 (Calypso Data upload (cgenome.net) and interpreted using compiled annotation files to delineate differences in abundance of Bifidobacterium, Lactobacillus and Clostridium between groups and time points. Data was filtered to remove rare taxa and normalised using TSS normalisation. Alpha diversity was assessed using both Shannon Index and the Chao1 and beta diversity using the Bray-Curtis Index. Results for abundance of Bifidobacterium, Lactobacillus and Clostridium were reported along with alpha and beta diversity.

#### Dietary Intake

Dietary intake was assessed using a three-day food diary which included two weekdays and one weekend-day to be completed in the week prior to the research visit. Mean daily values for energy (calories /day), macronutrients (g/day) and fibre (g/day) were calculated using Foodworks 9, Xyris software (www.xyris.com.au). Average daily serves of fruit, vegetables, wholegrains, legumes and yoghurt based on serve sizes as per national dietary guidelines (NHMRC, 2013) were calculated, to assess change in food groups known to be rich in prebiotics across the period of the intervention.

#### Adherence

Adherence was considered sufficient if at least 80% of the prescribed dose was taken. Parents returned unused supplement and a completed supplement diary to enable counting of dose consumed.

### Adverse Events

Parents were provided with the research team’s contact number and asked to contact the researchers in the event of any adverse reactions or if they had any concerns.

### Statistical Analysis

Linear mixed model (LMM) equations were used to determine between-group differences at follow-up for pQOL and for behavioural variables (mealtime behaviour, social behaviour) to account for missing data and to permit maximum data retention. For data missing at baseline, the baseline group mean was imputed to allow for inclusion in the model. A general linear model was also applied to pQOL data using baseline scores as a covariate to control for regression to the mean. Sensitivity analyses were carried out to evaluate the effect of outliers and to account for disproportionate groups at baseline. Where applicable, post hoc tests were carried out, using the Sidak correction. Effect size was determined using Cohen’s d, where 0.2 was considered small, 0.5 medium and 0.8 large (Cohen, 1988). An intention to treat analysis (ITT) was used for these variables (Parental QOL, Social behaviours and Mealtime behaviours). Mann Whitney U tests were used to compare change between-groups for GI symptoms, stool output (frequency and consistency), differential abundance of genera (Bifidobacterium, Clostridium and Lactobacillus), and alpha diversity of the microbiome. An ITT analysis was used for GI symptoms and stool output while a Per Protocol analysis (PP) was used for microbiome variables. Independent T-tests were used to compare between-group differences at follow-up for dietary energy, fibre, and macronutrients using a PP analysis, to control for differences in background diet. Statistical analysis was undertaken using SPSS (version 29) and Calypso (v8.84). Statistical significance was defined as *p* < 0.05.

## Results

**Fig. 2:**
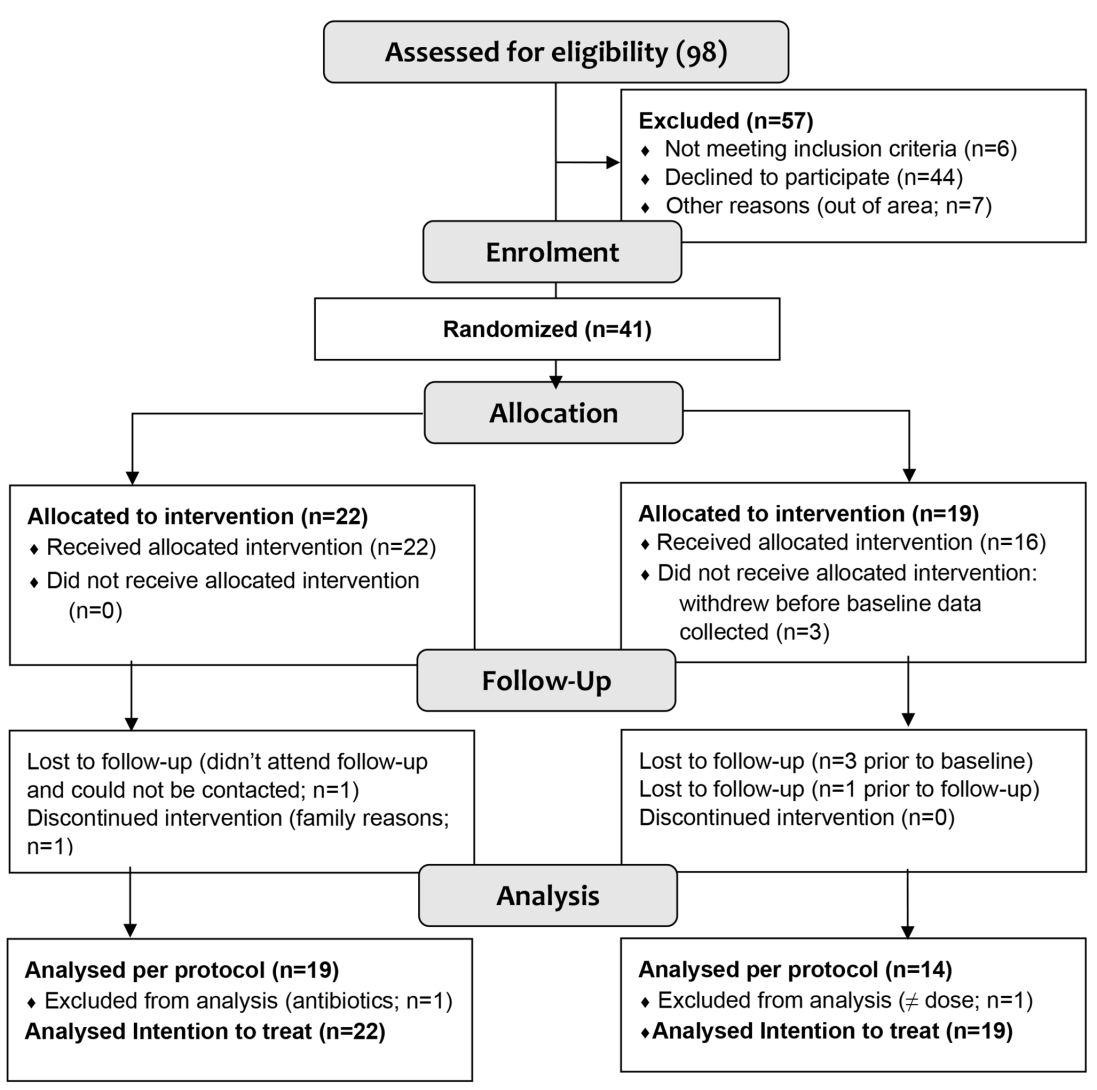
Consort flow diagram of enrolment to analysis

The consort diagram is shown in Fig. 2. Ninety-eight children were assessed for eligibility. Forty-one met the inclusion criteria, were enrolled and randomised to GOS or placebo (22 GOS; 19 placebo). Of these, three participants (siblings) from the placebo group withdrew from the study before collection of any baseline data, and before receiving the allocated supplement. These participants were included in the ITT analysis for pQOL, social, and mealtime behaviour using an imputed group mean. A further three participants withdrew (2 GOS; 1 placebo) during the study, which included two lost to follow-up (1 GOS; 1 placebo) and one withdrew for family reasons (1 GOS). In total, 35 parent-child dyads completed the study (20 GOS; 15 placebo), of which 33 participants (19 GOS; 14 placebo) were included in the per-protocol analysis. Two participants (1 GOS; 1 placebo) were siblings.

### Adherence and Adverse Events

Adherence to the dose for those that completed the intervention was similar across groups (GOS vs. placebo; 95 vs. 84%, *p* = 0.15), and overall, compliance with the study protocol was high (81%). No adverse effects were reported during the intervention phase.

### Demographic Characteristics

Demographic characteristics of children and parents are reported in Table 1. Groups were similar except for a greater prevalence of ADHD in children in the control group, and a higher proportion of children with more severe autism in the intervention (GOS) group.

**Table 1:** Demographic characteristics of participants and their carers in the intention-to-treat population

Child	Intervention (*n* = 22)	Control (*n* = 19)
Age, mean (± SD)	7.18 (1.36)	6.75 (1.65)
Ethnicity (Caucasion) n(%)	22 (100)	18 (95)
Sex (boys) n(%)	16 (68)	11 (58)
Autism severity Level 1 n(%)	5 (23)	9 (47)
Level 2 n(%)	14 (64)	9 (47)
Level 3 n(%)	3 (13)	1 (5)
Born via cesarian section n(%)	11 (50)	11 (58)*
Breastfed (any duration) n(%)	21 (95)	12 (63)*
ADHD n(%)	4 (18)	6 (31)
Anxiety (clinical) n(%)	1 (5)	3 (16)
Medication for ADHD n(%) Medication for Anxiety n(%) Medication for GI symptoms n(%)	4 (18) - -	5 (26) 1 (5) 2 (10)
**Parent / carer**		
Age in years, mean (± SD)	40.7 (5.5)	40.1 (4.7)*
Mother n(%)	21 (95)	18 (95)
Single parent n(%)	1 (4.5)	2 (10)
Tertiary educated n(%)	18 (82)	10 (62)*
Household income < AUD $80,000 n(%)	3 (14)	4 (25)*
Paid employment n(%)	18 (82)	10 (62)*
NDIS funding n(%)	15 (82)	7 (37)*
Other funding^a^ n(%)	12 (54)	12 (63)*
Partner / another child with autism n(%)	6 (27)	5 (26)

ADHD = attention deficit hyperactivity disorder, NDIS = National Disability Insurance Scheme, median annual household income QLD in 2016 = $73,000 AUD (ABS census of population and housing 2016), ^a^= other funding includes HCWA (Helping Children with Autism). *= 3 participants non-disclosed

### Parental Quality of Life (pQOL)

There were no significant group-differences at follow-up for pQOL for either component of the QoLA (Part A = typical QOL questions; Part B = impact of child’s behaviours on pQOL), (Table 2), however both groups improved from baseline for both Part A (*p* = 0.02) and Part B, (*p* < 0.001). Due to the small sample, effect size was also measured. A medium effect (*d* = 0.43) was detected for Part B, driven by a greater improvement in the GOS group (Table 2). There was no evidence that analyses were affected by regression to the mean when baseline scores were used as a covariate in a general linear model. There was also no effect of the intervention on pQOL when characteristics that may affect QOL such as work status and level of education were included as fixed effects in the Linear mixed model, (F[1,31] = 0.01, *p* = 0.97, Part A; F[1,31] = 1.01, *p* = 0.32, Part B.

**Table 2:** Parental quality of life scores for groups in the intention-to-treat population

Tool QoLA	Intervention Group (*n* = 22)		Control Group (*n* = 19)		*P* value^a^	Effect^d^
	Baseline	Follow-up	Baseline	Follow-up		
Part A	98 (91–105)	102 (95–109)	96 (88–103)	100 (92–108)	0.91	0.02
Part B	61 (55–69)	71 (64–77)	59 (52–66)	66 (58–73)	0.60	0.44

QoLA = Quality of Life Autism, data are mean (95% confidence interval), ^a^ from Linear Mixed Model between groups at follow-up, ^d^ effect size from Cohen’s d between groups at follow-up

### Social and Mealtime Child Behaviour

There were no group-differences at follow-up for the child’s social or mealtime behaviours (Table 3), however both groups improved significantly from baseline to follow-up for both social (GOS: 81 vs. 78; placebo: 82 vs. 79, p = < 0.001) and mealtime (GOS: 37 vs. 35; placebo: 39 vs. 36, p = < 0.001) behaviours respectively.

**Table 3:** Social behaviour and mealtime behaviour for groups in the intention-to-treat population

Tool	Intervention Group (*n* = 22)		Control Group (*n* = 19)		*P* value^a^	Effect^d^
	Baseline	Follow-up	Baseline	Follow-up		
SRS-2	81 (77–84)	78 (74–82)	84 (81–87)	79 (74–83)	0.42	0.12
BAMBI	39 (37–42)	35 (32–39)	42 (39–46)	36 (32–39)	0.17	0.03

SRS-2 = Social Responsiveness Scale, 2nd edition, BAMBI = Brief Autism Mealtime Behaviour Index, data are mean (95% confidence interval), ^a^ from Linear Mixed Model between groups at follow-up, ^d^ effect size from Cohen’s d between groups at follow-up

### Gastrointestinal Symptoms and Stool Output

There was no difference in change in overall GI symptoms between intervention and control groups, but there was a reduction in the percentage of children with severe GI symptoms (scores > 3) following GOS compared to the placebo. One-third of children in both groups had severe GI symptoms at baseline. At follow-up, prevalence of severe GI symptoms in the GOS group was lower compared to placebo (9% vs. 33%, Z = 1.63, *p* = 0.05) though this did not reach statistical significance. There was no difference in change in stool frequency or stool consistency between groups (Table 4).

**Table 4:** GI symptoms, stool frequency and stool consistency for groups in the intention-to-treat population

Tool	Intervention Group (*n* = 22)			Control Group (*n* = 19)			Change (Z)	*P* value^a^
	Baseline	Follow-up	Change	Baseline	Follow-up	Change		
**Total 6-GSI score**	2 (3.25)	2 (2.0)	-1 (3)	2 (3.0)	2 (3.0)	0 (4)	1.30	0.20
Severe GI symptoms (%)*	7 (32)	2 (9)	5 (23)	6 (33)	6 (33)	0 (0)	1.63	0.05
**Stool frequency / week**	5 (3)	4 (2)	-1(2)	7 (5)	7 (5)	0 (1.2)	1.20	0.23
**Consistency (Type)**	1.9 (2)	1.7 (2)	-0.1(1.7)	2.6 (1)	2.9 (1)	0 (2)	0.71	0.48

6-GSI = 6-item Gastrointestinal Symptom Index, severe symptoms (> 3), data are median (IQR) except for severity* where numbers are actual, ^a^ from Mann Whitney for difference in change between groups, consistency expressed as categories of the Bristol stool form scale

**Table 5:** Relative abundance of specific genera and alpha diversity for groups in the per-protocol population

Genus	Intervention group (*n* = 19)			Control group (*n* = 14)			Change (Z)	*P* value^a^
	Baseline	Follow-up	Change	Baseline	Follow-up	Change		
Bifidobacterium %	1.6 (8)	5.8 (8)	5.9 (11)	2.8 (8)	2.3 (6)	-0.4 (8)	-2.50	**0.001**
Clostridium %	2.7 (2)	2.1 (1)	0.05 (8)	1.6 (1)	1.6 (1)	0.1 (2)	0.07	0.96
Lactobacillus %	0.05(0)	0.04 (0)	0 (0.01)	0.02 (0)	0.02 (0)	0 (0.01)	-0.83	0.42
F:B ratio	2.3 (1.4)	2.0 (1.5)	0 (1.5)	1.6 (1.4)	1.4 (0.8)	-0.1 (1.7)	-0.95	0.36
**Diversity metric**								
Shannon Index	2.7 (0.3)	2.8 (0.4)	0 (0.2)	2.6 (0.4)	2.6 (0.3)	0.1 (0.3)	0.66	0.53
Chao 1	120 (7.5)	117 (6.4)	-3 (6.2)	117 (4.7)	119 (5.7)	1 (6.6)	2.20	**0.03**

Data are median (IQR), ^a^ from Mann Whitney for the difference in change between groups at follow-up, F:B ratio = Firmicutes:Bacteroidetes ratio

### Microbiome

**Fig. 3:**
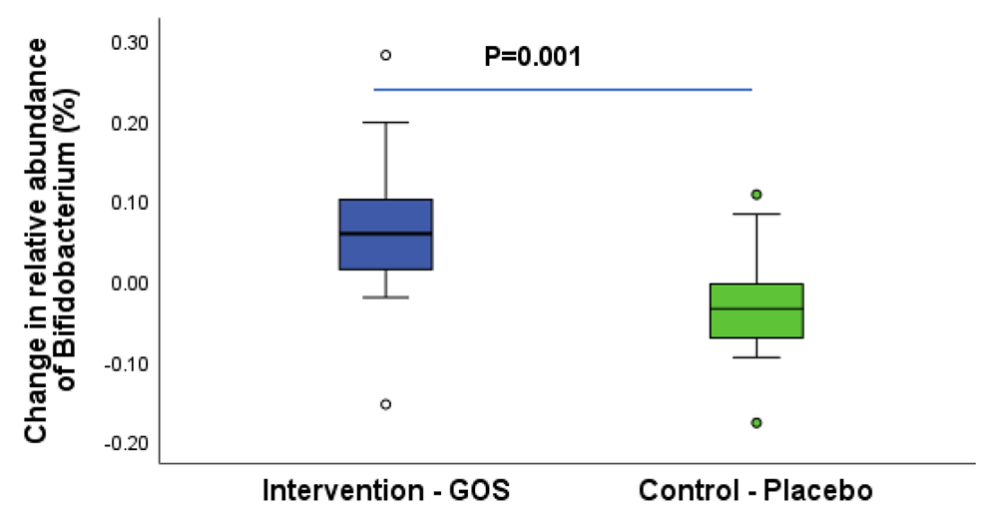
Change in relative abundance of Bifidobacterium for groups in the per-protocol population at follow-up. Data presented are median (central line), 25th and 75th centiles (box) and 1.5 interquartile range (whiskers) with data falling outside this range plotted as outliers

In total, 33 participants (19 intervention, 14 control) provided baseline and follow up stool samples. All samples were of good sequencing quality, providing approximately 100,000 reads and 3143 OTU’s. There was a significant difference between groups for change in relative abundance of Bifidobacterium, driven by a threefold increase in the intervention group (+ 5.9% vs. -0.4%; Z=-3.1, *p* = 0.001), (Fig. 3).

**Fig. 4:**
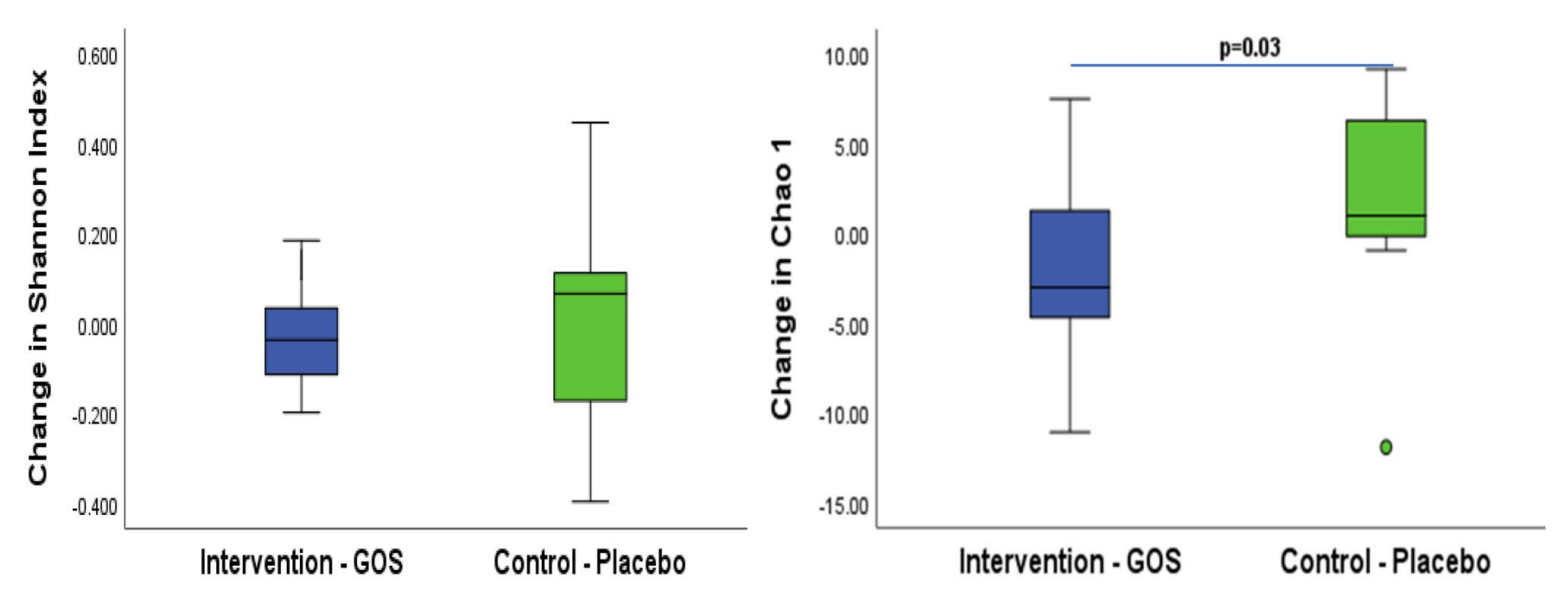
Change in alpha diversity for groups in the per-protocol population at follow-up. Data presented are median (central line), 25th and 75th centiles (box) and 1.5 interquartile range (whiskers) with data falling outside this range plotted as outliers

There was no difference between groups for change in other genera measured, (Table 5). Alpha diversity was similar across groups when assessed using the Shannon Index but there was a difference when assessed using the Chao1 driven by a reduction in alpha diversity in the GOS group and a small increase in the placebo group (-3 vs. 1, Z = 2.2, *p* = 0.03), (Fig. 4; Table 5).

**Fig. 5:**
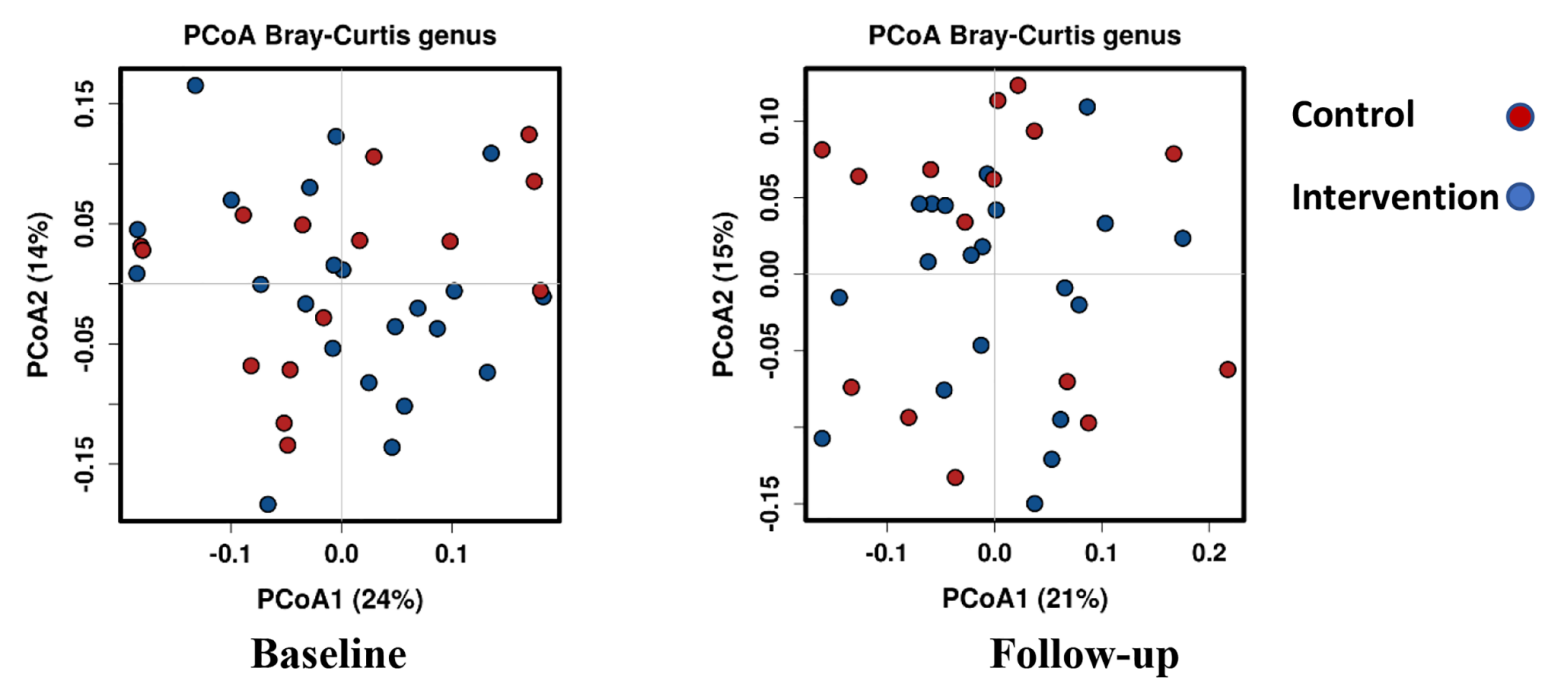
Bray curtis dissimilarity matrix: difference between groups at baseline and follow-up in the per-protocol population

No difference was seen between groups for beta diversity (Fig. 5).

### Dietary Intake

Dietary intake for energy, macronutrients and fibre was similar across groups at baseline and follow-up (Table 6). Servings of foods known to be a good source of prebiotics (fruit, vegetables, wholegrains, yoghurt, pulses) did not change significantly over the course of the intervention (Supplementary table S1) and it was assumed from the information provided, that dietary prebiotic intake did not change for either group over the course of the study.

**Table 6:** Daily energy, macronutrient and fibre intakes for groups in the per-protocol population

Nutrient	Intervention group (*n* = 19)		Control group (*n* = 14)		Baseline		Follow-up	
	Baseline	Follow-up	Baseline	Follow-up	F(df)^1^	*P* ^*1*^	F(df)^2^	*P* ^*2*^
Energy	6678 (2162)	6926 (1857)	7160 (1561)	6731 (1715)	1.83	0.65	0.01	0.52
Protein	60 (32)	69 (21)	69 (21)	65 (23)	0.03	0.23	0.81	0.48
Fat	58 (21)	63 (24)	70 (19)	62 (17)	0.66	0.62	0.40	0.55
CHO	198 (56)	195 (41)	194 (51)	172 (68)	4.70	0.80	0.51	0.70
Fibre	19 (7)	21 (4)	18 (11)	17 (6)	1.47	0.13	0.34	0.09

Data reported as median (IQR), energy reported in kJ/day, macronutrients and fibre reported in grams/day, *p* = from Independent T test for difference between groups (*p*^*1*^ at baseline; *p*^*2*^ at follow-up)

## Discussion

This trial investigated the effects of prebiotic GOS in children with autism and found no significant effect on pQOL, child social or mealtime behaviours, and only marginal effects on GI symptoms. Our hypothesis that GOS would improve a range of behaviours and symptoms associated with autism was not supported, even though there were significant bifidogenic effects on the microbiome in response to GOS, compared with placebo. Bifidobacterium increased three-fold in the intervention group, bringing levels in line with those seen in children without autism of a similar age (Berding et al., 2018) but this did not translate to a significant effect on clinical outcomes.

This is the first prebiotic intervention to investigate the downstream impact of modulating the microbiome of children with autism on parental QOL, so direct comparisons with other studies cannot be made. However, the use of an autism specific pQOL questionnaire enabled a closer look at how caring for a child with autism can impact pQOL and whether this can be influenced by the child’s gut microbiome. While there was not a significant difference between groups following supplementation, we observed a medium effect size (d = 0.44) for differences in pQOL related to the impact of child behaviours (QoLA, Part B), driven by greater improvements in the GOS group compared to placebo. Aberrant behaviours are known to be a major factor contributing to poorer QOL for parents of children with autism (McStay et al., 2014; Wang et al., 2018) and the positive effects seen are encouraging, highlighting a potential therapeutic value of prebiotic use in autism.

While previous interventions in children with autism have identified small but significant improvements in anti-social behaviours following supplementation with GOS (Grimaldi et al., 2018) or FOS and probiotic (Wang et al., 2020), or in aberrant behaviours related to irritability and stereotypy following supplementation with bovine colostrum (Sanctuary et al., 2019), we did not see any differences between groups at follow-up for social behaviours. This contrast in findings might be explained by the difference in study design (prebiotic vs. synbiotic, cross-over vs. parallel arm) or length of intervention. For example, Wang et al. (2020) saw improvements in anti-social behaviours but only after 12 weeks of supplementation, where improvements in GI symptoms were seen within six weeks. It should be noted that at the time of this study design, there were no published prebiotic interventions in children with autism to guide time frames or choice of prebiotic. In addition, variation in assessment tools and outcomes measured may contribute to differing results as the SRS-2 focuses on aspects of social responsiveness and repetitive behaviours while other tools such as the ATEC (Rimland & Edelson, 1999) and ABC (Krug et al., 1980) include broader constructs of physical health and irritability, and hyperactivity.

We observed significant improvements in social and mealtime behaviours in both groups at follow-up, which made delineation of prebiotic effects difficult. Access to a dietitian with experience in autism was deemed an ethical addition and incentive to participate, as well as replicating a typical dietetic setting, but this may have biased the results. Research has shown that perceived professional support is associated with lower levels of care-giver stress in this population (Goedeke et al., 2019), and that lack of support in general correlates strongly to lower pQOL in parents of children with autism (Vasilopoulou & Nisbet, 2016).

Supplementation with GOS led to no improvements in GI symptoms, stool frequency or stool consistency compared with placebo. Although previous research suggests improvements in diarrhea and pain on stooling, there was no reported benefit in constipation (Sanctuary et al., 2019), despite studies observing softer stools and reduced straining in children (without autism) with constipation (Beleli et al., 2015; Closa-Monasterolo et al., 2017). This apparent contrast in findings might be explained by a heterogeneous pathophysiology of GI symptoms in autism. Although no overall improvement in GI symptoms was seen, of those reporting severe GI symptoms at baseline, fewer children taking GOS reported severe symptoms at follow-up compared to the placebo. The medium effect size observed between groups for improvements in severe GI symptoms was driven by greater improvements in bloating and abdominal pain in the GOS group. These differences could be seen as clinically meaningful (albeit not statistically significant) and potential benefit of prebiotics for children presenting with more severe GI symptoms warrants further investigation.

With regards to the microbiome, GOS supplementation led to a significant increase in Bifidobacterium abundance compared with placebo. This is line with previous research showing GOS supplementation at similar doses can lead to substantial enrichment of this taxa (Grimaldi et al., 2017; Silk et al., 2009). While the data demonstrate that the relative abundance of Lactobacillus and Clostridium did not change in response to GOS, despite GOS being a proven substrate for Lactobacillus (Hernandez-Hernandez et al., 2012), changes may have occurred at lower taxonomic levels not captured by 16 S sequencing. Bifidobacterium are highly competitive for GOS (Macfarlane et al., 2008) and may have simply outcompeted other genera, but this is impossible to determine without analysis at the level of species. While no difference in Shannon diversity was observed, there was a significant difference in Chao1 with a reduction in diversity in the GOS group, mostly likely explained by the increase in Bifidobacterium at the expense of rare taxa, but again, analysis at the level of species is needed to confirm this supposition.

There were many strengths of the study. We recruited a community sample representative of children with varying levels of autism and included measures of QOL and behaviour that have been validated in the autistic population. Microbiome was measured to confirm the impact of the microbial composition, and dietary intake was carefully assessed to ensure no confounding by background diet. Encouragingly, the supplement was not only well tolerated with no reported adverse effects (even in those with severe baseline GI symptoms), but also well accepted in a cohort notoriously hypersensitive to change in sensory aspects of food. The high rate of adherence suggests that this is a feasible treatment for use in practice and future research.

However, our study was small and despite rigorous methodology and the use of validated, autism specific tools, we were not able to capture any subtleties in behaviour change. Children included in the study had been previously diagnosed with ASD and admission depended on sighting of their letter of diagnosis or funding for autism. No further diagnostic assessment was undertaken upon enrolment which is a limitation of the study. The inclusion criteria were voluntarily broad to represent the paediatric autistic community, but this made it difficult to determine clear effects of the prebiotic. With this being a pilot trial, no statistical adjustments were made for multiple comparisons thus the possibility of chance findings needs to be considered. Larger trials that recruit a more homogenous sample with severe presentations of variables of interest are required to evaluate the clinical effects of GOS. Finally, including dietetic counselling may have had confounding effects and separate arms should be considered, particularly as strong placebo effects are common in autism interventions (Siafis et al., 2020).

Future studies should include a qualitative component to evaluate acceptability of the intervention and assist with the interpretation of findings. A focus on parental wellbeing or parental stress which has been shown to be influenced by child behaviours (Giovagnoli et al., 2015) might also prove more realistic over a short time frame compared to the complex construct of QOL. Finally, a combination of prebiotics may elicit more widespread change in the microbiome. For example, the addition of FOS has the potential to enrich other commensal microorganisms such as *Faecalibacterium* which produces butyrate, important to immunomodulation and maintenance of gut barrier function (Cryan et al., 2019; Ferreira-Halder et al., 2017). Immune function and the gut barrier have both been proposed as factors contributing to GI symptoms and aberrant behaviours in autism (Adams et al., 2011; Robinson-Agramonte et al., 2022).

## Conclusion

While the gut microbiome is increasingly being investigated for potential effects on state of mind and behaviour, it has proven difficult to consistently translate the convincing results seen in preclinical studies to humans. This six-week intervention using prebiotic GOS to supplement the diet of children with autism was well tolerated and significantly increased the abundance of *Bifidobacterium.* However, the relatively small sample size and heterogeneous symptom presentation made it difficult to differentiate prebiotic effects. A medium effect size detected for improvements in pQOL and GI symptoms following supplementation with GOS suggests the need for a larger intervention to confirm whether there is as role for prebiotic supplementation in autism. Although there was no significant difference in outcomes between groups, the high rates of compliance, absence of side-effects and medium effects detected were encouraging and warrant further investigation.

## Data Availability

The dataset supporting the conclusions of this article in the QUT Datafinder repository: Palmer, Jacqui; van der Pols, Jolieke; Sullivan, Karen; Staudacher, Heidi; Byrne, Rebecca; (2023): Prebiotic supplement use, the gut microbiome and behaviour change in children with autism spectrum disorder (ASD). Queensland University of Technology. (Dataset) 10.25912/RDF_1683174974223.
